# Fungus *Pichia kudriavzevii* XTY1 and heterotrophic nitrifying bacterium *Enterobacter asburiae* GS2 cannot efficiently transform organic nitrogen *via* hydroxylamine and nitrite

**DOI:** 10.3389/fmicb.2022.1038599

**Published:** 2022-12-08

**Authors:** Tianyue Xu, Simeng Song, Baihui Ren, Jiahuan Li, Jiyun Yang, Long Bai, Zhongyun Piao

**Affiliations:** College of Horticulture, Shenyang Agricultural University, Shenyang, China

**Keywords:** organic nitrogen transformation, fungi, heterotrophic nitrifying bacteria, heterotrophic nitrification, ^15^N labeling

## Abstract

Heterotrophic nitrification is a process of organic nitrogen degradation completed by the participation of heterotrophic nitrifying microorganisms, which can accelerate the nitrogen transformation process. However, the current research mainly focuses on heterotrophic nitrifying bacteria and their ammonium degradation capacities. And there is little accumulation of research on fungi, the main force of heterotrophic nitrification, and their capacities to transform organic nitrogen. In this study, novel heterotrophic nitrifying fungus (XTY1) and bacterium (GS2) were screened and isolated from upland soil, and the strains were identified and registered through GenBank comparison. After 24 h single nitrogen source tests and ^15^N labeling tests, we compared and preliminarily determined the heterotrophic nitrification capacities and pathways of the two strains. The results showed that XTY1 and GS2 had different transformation capacities to different nitrogen substrates and could efficiently transform organic nitrogen. However, the transformation capacity of XTY1 to ammonium was much lower than that of GS2. The two strains did not pass through NH_2_OH and NO_2_^−^ during the heterotrophic nitrification of organic nitrogen, and mainly generated intracellular nitrogen and low N_2_O. Other novel organic nitrogen metabolism pathways may be existed, but they remain to be further validated.

## Highlights

- Two strains of heterotrophic nitrifying microorganisms were screened and isolated from soil. The fungus was named *Pichia kudriavzevii* XTY1, and the bacterium was named *Enterobacter asburiae* GS2. The gene sequences were 471 bp and 1,385 bp in length, and the GenBank registration numbers were MW132885 and MW126534, respectively.
- XTY1 and GS2 could efficiently utilize organic nitrogen for heterotrophic nitrification with transformation rates of 83.6 and 82.8%, respectively. However, the transformation capacities of inorganic N were different, and the transformation rate of ammonium by XTY1 (41.3%) was much lower than that of GS2 (82.0%).
- During the heterotrophic nitrification of organic nitrogen by XTY1 and GS2, no hydroxylamine and nitrite were produced, more than 60% of the organic nitrogen was generated into intracellular nitrogen, and less than 0.3% was generated into N_2_O. Unlike previous findings, there may be novel pathways.

## Introduction

Heterotrophic nitrifying microorganisms transform soil organic nitrogen (N_org_) directly to nitrate (NO_3_^−^) without ammonification, thereby accelerating the process of nitrogen transformation. Heterotrophic nitrification is considered to be a new pathway in the nitrogen transformation process, which launched the research on heterotrophic nitrifying microorganisms (Robertson and Kuenen, 1983; Serna and Pomares, 1992; Brierley and Wood, 2001). Over the last three decades, many researchers have identified a variety of heterotrophic nitrifying microorganisms in various habitats, and described their ammonium (NH_4_^+^) transformation capacities and metabolite characteristics (Pedersen et al., 1999; Joo et al., 2005; Schmidt et al., 2011; Zhang et al., 2014; Bai et al., 2018; Yu et al., 2020; Zhang et al., 2020), but little is known about the N_org_ transformation pathways and characteristics.

At the same time, the studies on fungi (Boer and Kowalchuk, 2001; Liu et al., 2016), the main undertaker of nitrification, are far fewer than that on heterotrophic nitrifying bacteria. These studies have only focused on the screening of fungi, and did not explore their nitrogen (N) transformation in depth. Therefore, studying heterotrophic nitrifying fungi and their capacities to transform N_org_ are beneficial to deepen researchers’ understanding of heterotrophic nitrifying microorganisms.

Different heterotrophic nitrifying microorganisms have different nitrogen transformation pathways. For example, *Alcaligenes faecalis* No. 4 and *Acinetobacter* sp. JR1 transformed NH_4_^+^ first to hydroxylamine (NH_2_OH), then to nitrite (NO_2_^−^), and finally to NO_3_^−^ *via* nitrification (Joo et al., 2005; Yang et al., 2019). By comparison, *Alcaligenes faecalis* NR transformed NH_4_^+^ to NH_2_OH and then directly to nitrous oxide (N_2_O) and nitrogen gas (N_2_) without passing through NO_2_^−^ and NO_3_^−^ (Zhao et al., 2012). *Agrobacterium* sp. LAD9 isolated by Chen and Ni (2012) and *Alcaligenes ammonioxydans* sp. HO-1 isolated by Wu et al. (2021) denitrified to N_2_ after transformation of NH_4_^+^ to NO_2_^−^. In contrast, *Pseudomonas putida* Y-9 directly transformed NH_4_^+^ to nitric oxide (NO) and then to N_2_O, and no other intermediates were found (Huang et al., 2019). Therefore, we also need to track the metabolites and pathways of the isolated strains to understand the nitrogen transformation method of each microorganism.

Under the background of global changes such as atmospheric nitrogen deposition and atmospheric warming, screening and isolating heterotrophic nitrifying strains from soil and understanding the transformation capacities of strains to organic nitrogen are of great significance for preventing soil pollution and reducing greenhouse gas emissions, etc. Subsequently, our research group screened and isolated fungus and heterotrophic nitrifying bacterium with high organic nitrogen transformation capacities in the soil of the upland experimental area, and identified their species after comparing with the GenBank. We inoculated the strains with N_org_ (glycine), NH_4_^+^, NO_3_^−^, potential intermediate products (NH_2_OH and NO_2_^−^) as the sole nitrogen sources of the medium for 24 h incubation tests, respectively, and determined the heterotrophic nitrification and aerobic denitrification capacities and intermediate products of the two strains. We then inoculated the strains with N_org_ (glycine), NH_4_^+^, NO_3_^−^ as the sole nitrogen sources of the medium, combined with ^15^N labeling method to determine the N_2_O and N_2_ emissions of heterotrophic nitrification and aerobic denitrification processes, to preliminarily understand and analyze the N_org_ transformation pathways of two strains. Finally, it provided potential strains for heterotrophic nitrification and further enriched the nitrogen transformation pathways of heterotrophic nitrifying microorganisms. We hypothesized that: (i) Do heterotrophic nitrifying microorganisms have both autotrophic nitrification and heterotrophic nitrification capabilities? Is there a difference between the two? (ii) Are the intermediates of heterotrophic nitrification processes the same as those of conventional autotrophic nitrification processes?

## Materials and methods

### Media

The fungal Screening medium (Bengal Red) contained the following components (per liter): 5.0 g peptone, 10.0 g glucose, 1.0 g KH_2_PO_4_, 0.5 g MgSO_4_·7H_2_O, 0.03 g Bengal Red, and 0.1 g chloramphenicol. The bacterial enrichment medium (LB) contained the following components (per liter): 3.0 g beef paste, 10.0 g peptone, 5.0 g NaCl. The bacterial isolation and purification medium contained the following components (per liter): 0.5 g glycine, 4.0 g trisodium citrate, 1.6 g NaCl, 8.2 g KH_2_PO_4_, 0.5 g MgSO_4_·7H_2_O, 0.01 g FeSO_4_·7H_2_O, 0.5 g KCl. The basal medium contained the following components (per liter): 1.0 g KH_2_PO_4_, 0.5 g MgSO_4_·7H_2_O, 0.03 g FeSO_4_·7H_2_O, 0.12 g NaCl, 0.01 g CaCl_2_, and 0.1 g chloramphenicol (Bacterial medium was not added). Different nitrogen and carbon sources were added to the basal medium depending on the experiment (Tables 1 and 2). The initial pH of the medium was adjusted to 7.2 ± 0.2 using 0.1 M HCl or 0.1 M NaOH. The solid medium was supplemented with 1.3–1.5% agar. All media were autoclaved at 121°C for 20 min before use. All chemicals were of analytical grade and were weighed on an electronic scale (precision 1 × 10^−4^).

**Table 1:** List of carbon and nitrogen sources added to basal medium (BM) for nitrification and denitrification by XTY1.

Component	Nitrogen and carbon amount (g·L^−1^)				
	BM-1	BM-2	BM-3	BM-4	BM-5
C_2_H_5_NO_2_	0.20	–	–	–	–
(NH_4_)_2_SO_4_	–	0.94	–	–	–
NH_2_OḤHCl	–	–	0.50	–	–
NaNO_2_	–	–	–	0.49	–
KNO_3_	–	–	–	–	1.44
C_6_H_5_Na_3_O_7_·2H_2_O	1.26	8.14	4.11	4.06	8.15

**Table 2:** List of carbon and nitrogen sources added to basal medium (BM) for nitrification and denitrification by GS2.

Component	Nitrogen and carbon amount (g·L^−1^)				
	BM-6	BM-7	BM-8	BM-9	BM-10
C_2_H_5_NO_2_	0.20	–	–	–	–
(NH_4_)_2_SO_4_	–	0.94	–	–	–
NH_2_OḤHCl	–	–	0.50	–	–
NaNO_2_	–	–	–	0.49	–
KNO_3_	–	–	–	–	1.44
C_6_H_5_Na_3_O_7_·2H_2_O	2.02	12.22	6.17	6.09	12.23

### Screening and identification

Soil samples were collected from upland at the Baicao Garden Teaching and Research Base of Shenyang Agricultural University, Shenyang, Liaoning Province, China (41.83° N, 123.57° E). The site was a perennial grassland planted with *Poa annua* L. (species of excellence) without fertilization treatment and belongs to a temperate semi-humid continental climate zone, with an annual average precipitation of 580 mm and average temperate of 24.8°C from April to October. The brown soil texture is 27.2% sand, 50.2% silt and 22.6% clay. The soil pH was 5.74, total carbon (C) was 32.11 g·kg^−1^, total phosphorus was 1.42 g·kg^−1^, total potassium was 30.29 g·kg^−1^, total N was 1.22 g·kg^−1^, NH_4_^+^ was 13.70 mg·kg^−1^ and NO_3_^−^ was 5.53 mg·kg^−1^, available phosphorus was 13.40 mg·kg^−1^, available potassium was 132.34 mg·kg^−1^.

Three sites with good growth and no baldness were searched for in the sampling area to take 0–10 cm soil samples using the three-point method. Ten gram of the soil sample was placed in a 250-mL conical flask containing 90 ml sterile water and incubated in a rotary shaker at 25°C and 150 rpm for 1 h. After standing for 2 h, 2 ml of the suspension was added to 250-mL conical flasks containing 98 ml fungal screening medium and bacterial enrichment medium and incubated at 35°C and 150 rpm for 2 d. For bacterial screening, an additional 2 ml suspension of enrichment medium was added to a 250-mL conical flask containing 98 ml of bacterial screening medium and incubated at 35°C at 150 rpm for 1 d. The above culture medium inoculation processes were repeated three times.

We applied 200 μl of the suspensions on the respective solid screening medium, and the strains were isolated by plate streaking until a single colony could be picked and inoculated in 250-mL conical flasks containing 100 ml BM-1, 6 medium (Tables 1, 2). These cultures were shaken at a constant temperature for 24 h. The glycine concentration in the medium was measured, and the fungal and bacterial strains with the highest transformation rate were selected for further experiments. The strains were inoculated in 250-mL conical flasks containing 98 ml respective screening medium and shaken at 35°C until the culture reached logarithmic phase, whereby it was mixed 1:1 with 30% glycerol and placed at −80°C for long-term storage.

Scanning electron microscopy and transmission electron microscopy (bacteria only) were used to observe the fungal and bacterial cell morphology. ITS rRNA and 16S rDNA were determined by polymerase chain reaction (PCR) amplification. Fungal primers ITS1 (TCCGTAGGTGAACCTGCGG) and ITS4 (TCCTCCGTTATTGATATATGC), bacterial universal primers 27F (5’-AGAGTTTGATCCTGGCTCAG-3′) and 1492R (5’-CTACGGCTACCTTGTTACGA-3′), these primer synthesis and culture identification processes were completed by Sangon Biotech (Shanghai, China). The PCR thermal program of the fungus was conducted according to the following cycle profile: pre-denaturation at 95°C for 5 min, 35 cycles of denaturation at 95°C for 30 s, annealing at 58°C for 30 s, and extension at 72°C for 1 min and 30 s in a thermal cycler. After the last cycle, incubate for another 7 min at 72°C to allow the extension to complete. The PCR thermal program for bacteria was the same as above, except for 1 min at 72°C in a thermal cycler. Finally, BLAST analysis of the National Center for Biotechnology Information was used for sequence analysis and homology comparison to determine fungi and bacterial genera.

### Heterotrophic nitrification and aerobic denitrification capacity

Firstly, the optimal conditions of heterotrophic nitrifying strains were screened by orthogonal experiment. The results showed that the fungal transformation rate was highest with trisodium citrate as the carbon source, 200 mg·L^−1^ initial glycine, and the ratio of total carbon to total nitrogen in the culture medium (C/N ratio) of 10, 35°C, and 130 rpm rotating speed. The bacterial transformation rate was highest under culture conditions with trisodium citrate as the carbon source, 200 mg·L^−1^ initial glycine, and C/N ratio of 15, 35°C, and 160 rpm. Therefore, subsequent experiments were conducted under these conditions. Researches with soil revealed that free amino acids were an important contributor to the soil soluble N_org_ pool, with glycine, aspartic acid, and glutamic acid accounting for the largest fraction of N_org_ (Paul and Williams, 2005; Streeter et al., 2015). Hence, glycine was chosen as the organic nitrogen source for heterotrophic nitrification in this study.

Heterotrophic nitrification and aerobic denitrification of the candidate fungal and bacterial strains were determined using N_org_ (glycine), (NH_4_)_2_SO_4_, and KNO_3_ as sole nitrogen sources. Two milliliters of fungal and bacterial sample solutions stored at −80°C were inoculated in 250-mL conical flasks containing 98 ml BM-1, 6 medium and incubated at 35°C and 130 (160) rpm for 24 h to activate the strains. The pre-cultures were centrifuged at 4000 G and 4°C for 2 min, and then washed three times with sterile water. Next, 2 ml of the sterile water-rinsed solutions was inoculated in 250-mL conical flasks containing 98 ml BM-1, 2, 5 and BM-6, 7, 10 medium (Tables 1, 2), and incubated at 130 (160) rpm and 35°C for 24 h. Each sample was assayed in triplicates, and medium without inoculum was used as the control. Samples were taken at 2 h intervals, centrifuged at 11000 G for 10 min and the supernatants were taken to determine glycine, NH_4_^+^, NO_2_^−^, NH_2_OH, NO_3_^−^, intracellular nitrogen, and total nitrogen concentrations. as well as cell growth by measuring optical density at a 600 nm wavelength (OD_600_) without centrifuging the samples.

### Utilization of hydroxylamine and nitrite

Pre-treatment of the activated sample solution was as described in section 2.3. The sterile water-rinsed fungal and bacterial solutions (2 ml) were inoculated into 250-mL conical flasks containing 98 ml BM-3, 4 and BM-8, 9 medium (Tables 1, 2) with NH_2_OḤHCl and NaNO_2_ as sole nitrogen sources, and incubated at 130 (160) rpm and 35°C for 24 h. Three replicates were performed. Samples were taken every 4 h to determine NH_4_^+^, NO_2_^−^, NH_2_OH, NO_3_^−^, intracellular nitrogen, and total nitrogen concentrations, as well as OD_600_.

### Gaseous product determination

N_2_O and N_2_ produced during heterotrophic nitrification and aerobic denitrification were determined using a single ^15^N-labeled nitrogen source (99 atom%). The activated sample solutions (2 ml) were inoculated in 250-mL airtight silk-mouthed bottles containing 98 ml BM-1, 2, 5 and BM-6, 7, 10 media labeled with ^15^N. The space above the liquid was filled with ambient air and flasks were shaken at 35°C and 130 (160) rpm for 24 h. Then, 2 ml of the gas above each sample was extracted for the determination of N_2_ and N_2_O content. Three replicates were performed. These analyses were conducted at the Shenyang Institute of Applied Ecology, Chinese Academy of Sciences.

### Analytical methods and calculations

Cell growth was determined by measuring OD_600_ on an ultraviolet (UV) spectrophotometer. Glycine was determined using the ninhydrin colorimetric method, NH_4_^+^ using the indophenol blue colorimetric method, NH_2_OH by the Frear and Burrell (1955) method, and NO_2_^−^ by the N-(1-naphthyl)-ethylenediamine method. Nitrate and total nitrogen were determined by UV spectrophotometry (Clescerl, 1998). Intracellular nitrogen was measured by differential subtraction, whereby the total nitrogen concentration after centrifugation was subtracted from that before centrifugation (Rout et al., 2017). N_2_O and N_2_ were determined by mass spectrometry.

The MEGA 5.0 software was used to build the phylogenetic tree. Data analysis was performed using Microsoft Excel and the SPSS 22 software. Graphs were plotted using SigmaPlot 10.0, and results were presented as means ± standard errors.

## Results and discussion

### Screening and identification

**Figure 1:**
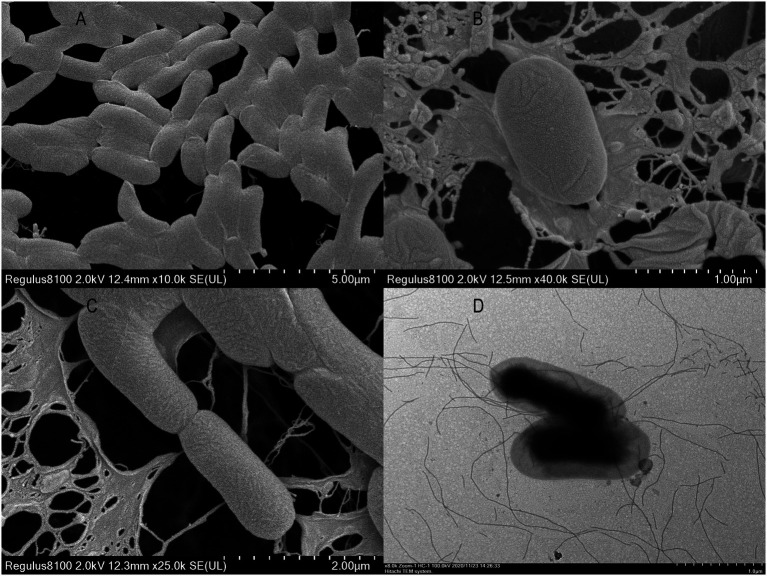
Electron microscopy image of XTY1 **(A, C)** and GS2 **(B, D)**.

**Figure 2:**
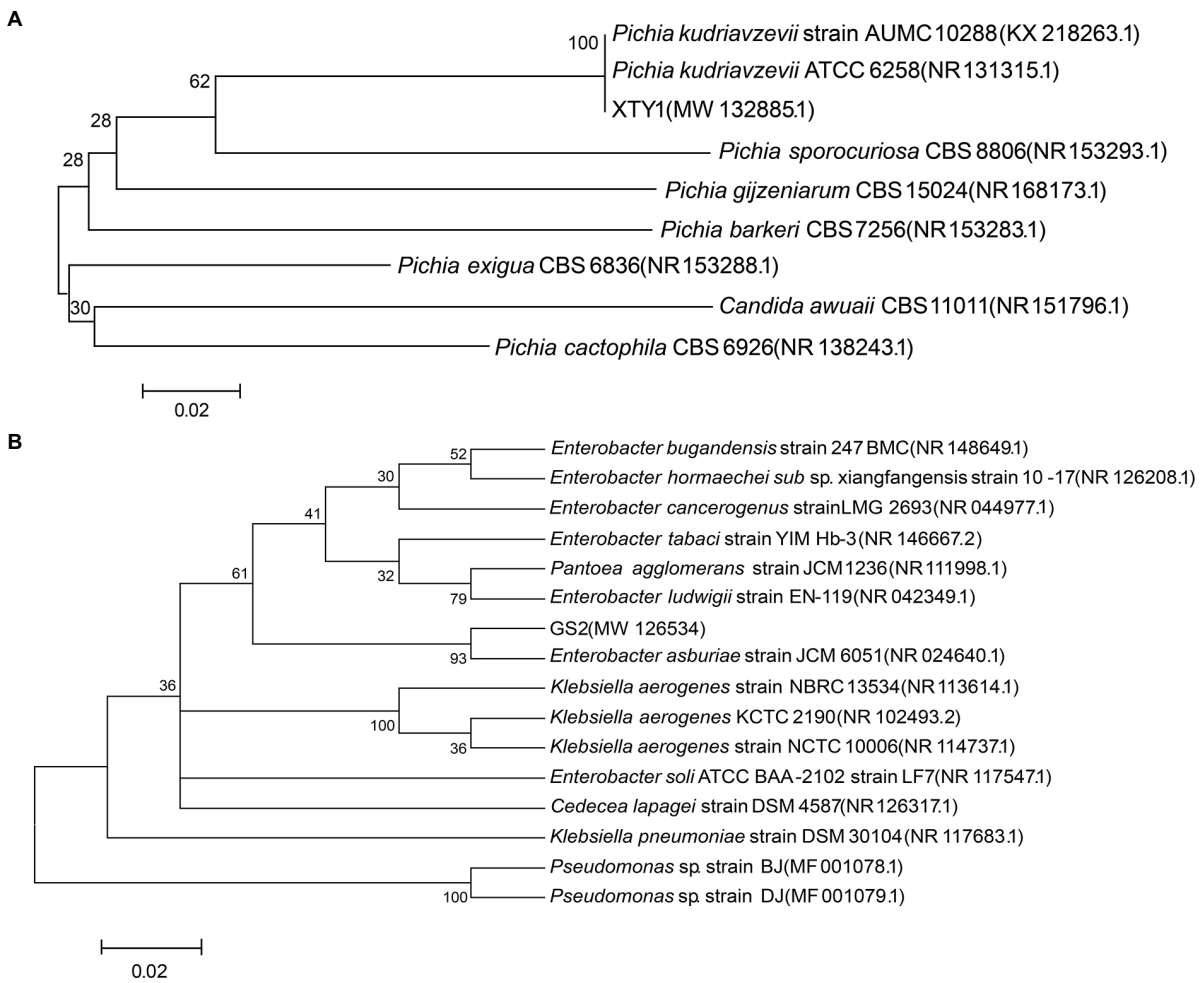
Phylogenetic tree of XTY1 **(A)** and GS2 **(B)**. Node support values are presented, the scale bar defines the branch length.

Three strains of heterotrophic nitrifying fungi were screened on solid Bengal Red medium, revealing glycine transformation rates of 30.1, 50.2, and 83.8%. The fungus with the strongest transformation capacity was named XTY1 and was selected for subsequent experiments. Its colonies appeared round, creamy white, with a smooth and moist surface, whereas cells were elongated and (2.4–2.5) μm × 0.8 μm in length (Figures 1A,C). To identify the genus of the strain, a phylogenetic tree constructed after PCR amplification of a 471 bp fragment revealed that the candidate strain had 99% homology with *Pichia kudriavzevii* (Figure 2A). Hence, the strain was named *Pichia kudriavzevii* XTY1, referred to herein as XTY1. The sequence was deposited in the GenBank database under registration number MW132885.

Two strains of heterotrophic nitrifying bacteria were also screened, with glycine transformation rates of 57.0 and 82.7%. The second bacterium with stronger transformation capacity was named GS2 and used for the follow-up experiment. The colonies were round, off-white and translucent with smooth and moist surface and regular edges. The cells were short rod-shaped with flagella, and the size was about (1.3–1.6) μm × (0.6–0.8) μm (Figures 1B,D). A fragment of 1,385 bp was amplified by PCR, and the comparison revealed that the candidate strain had 99% homology with *Enterobacter asburiae* (Figure 2B). The strain was finally named *Enterobacter asburiae* GS2 (GS2 for short) and was registered in the GenBank database under registration number MW126534.

### Heterotrophic nitrification and aerobic denitrification capacity

**Figure 3:**
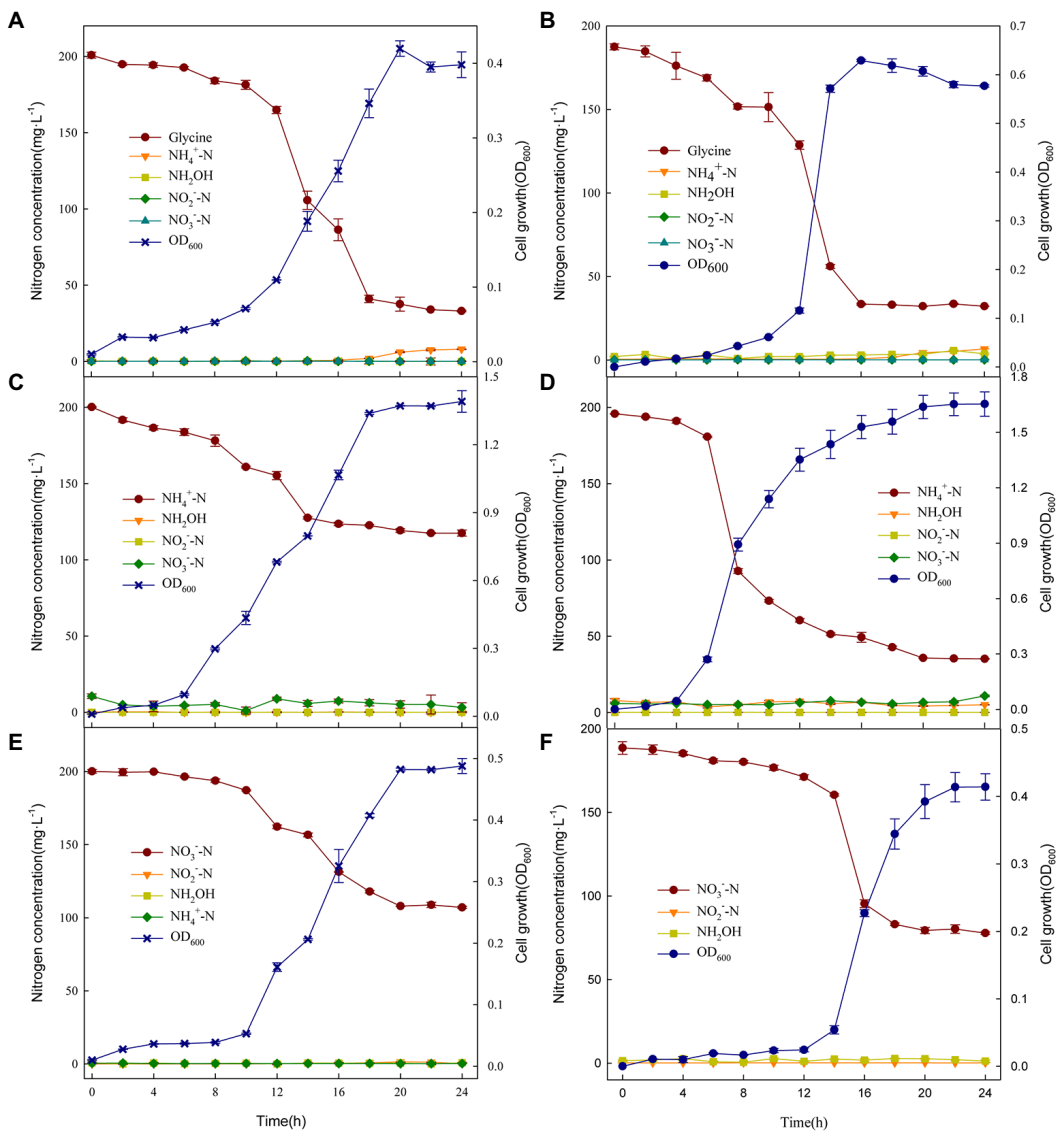
Changes in nitrogen compounds and growth of XTY1 and GS2 when glycine **(A, B)**, (NH_4_)_2_SO_4_ **(C, D)**, or KNO_3_ **(E, F)** were used as sole nitrogen sources under aerobic conditions.

As shown in Figure 3A, fungus XTY1 grown on glycine displayed a lag phase of 4 h, and started the logarithmic phase after 12 h, reaching maximum growth (D_600_ = 0.42) after 20 h and entered senescence thereafter. At the same time, glycine decreased rapidly after 12 h, reached the maximum transformation rate of 29.60 mg·L^−1^·h^−1^ at 14 h, and stabilized after 20 h. This decreasing trend was consistent with the growth trend of XTY1, indicating that the strain could utilize glycine for growth. After 20 h, the cell growth decreased slightly, while the NH_4_^+^ concentration increased, which can be explained by cellular transformation, possibly due to the release of NH_4_^+^ by senescent death of the strain (Chen et al., 2014; Li et al., 2015). with the fungus XTY1 and the bacterium GS2 (Figure 3B) were not significantly different in their capacity to transform glycine, and the concentrations of nitrogen-containing intermediates NH_4_^+^, NH_2_OH, NO_2_^−^, and NO_3_^−^ did not change significantly during the 24 h incubation period.

As shown in Figure 3C, growth of XTY1 on (NH_4_)_2_SO_4_ as the nitrogen source led to no lag phase and stabilization after 18 h, with a maximum OD_600_ of 1.39. The NH_4_^+^ concentration decreased from 200.15 mg·L^−1^ to 117.56 mg·L^−1^, with a maximum transformation rate of 13.88 mg·L^−1^·h^−1^, indicating that NH_4_^+^ could be used for both growth and nitrogen transformation. At 0 h, a small accumulation of NO_3_^−^ was observed, probably due to oxidation of NH_4_^+^ followed by its gradual reduction during the subsequent incubation (Li et al., 2015). Compared to GS2 (Figure 3D), the fungal heterotrophic nitrification of ammonium was very low. No significant changes in other intermediates were detected, suggesting that even though the fungi play a major role in heterotrophic nitrification (Zhang et al., 2011), the heterotrophic nitrifying fungi were much less capable of transformation than the bacteria. There were small fluctuations in NO_3_^−^ throughout the incubation period of both strains, probably due to the transformation of NH_4_^+^ to NO_3_^−^ by the strains.

Incubation with KNO_3_ as the sole nitrogen source resulted in a long lag phase for XTY1, which entered the logarithmic phase only after 8 h, when the concentration of NO_3_^−^ decreased sharply, dropping to 107.07 mg·L^−1^ at 24 h (Figure 3E). This suggests that the strain could use NO_3_^−^ for aerobic denitrification. Compared with GS2 (Figure 3F), XTY1 showed similar aerobic denitrification of nitrate, with no significant changes in the content of other nitrogenous substances throughout the incubation.

### Study on heterotrophic nitrification intermediates and pathways of strains

**Figure 4:**
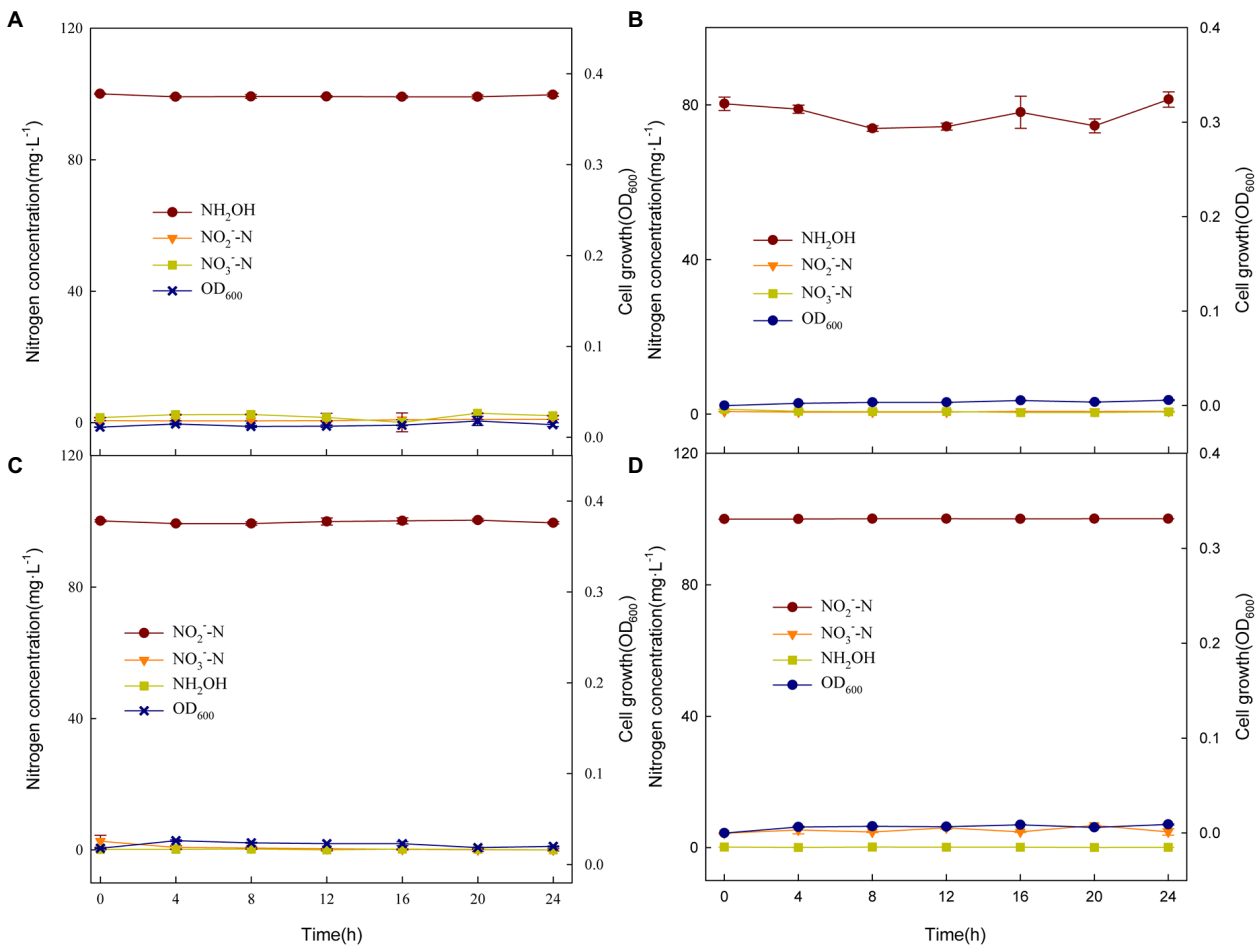
Changes in nitrogen compounds and growth of XTY1 and GS2 when NH_2_OḤHCl **(A, B)** or NaNO_2_ **(C, D)** were used as sole nitrogen sources under aerobic conditions.

At present, researchers believe that hydroxylamine and nitrite are the most likely intermediates for heterotrophic nitrifying strains to transform nitrogen sources, such as *Pseudomonas bauzanensis* DN13-1 (Zhang et al., 2020) and *Ochrobactrum anthropi* HND19 (Ren et al., 2021). Therefore, NH_2_OḤHCl (Figures 4A,B) and NaNO_2_ (Figures 4C,D) were used as the sole nitrogen sources for XTY1 and GS2 culture experiments. However, the experiments showed that the two strains did not grow and the nitrogen concentration did not change over 24 h. This result confirmed that NH_2_OH and NO_2_^−^ were not detected during the incubation process as a result of non-production and not by transformation. There might be new metabolites in the nitrogen transformation process. Therefore, the metabolites of XTY1 and GS2 with glycine as nitrogen source (BM-1, 6 medium) for 16 h were preliminarily detected. We compared the nitrogen-containing substances in the culture medium with glycine transformation products retrieved from Glycine, serine and threonine metabolism^1^ in KEGG official website, such as Glyoxylic acid (C00048), S-Amino-methyldihydrolipoylprotein (C01242), Lipoylprotein (C02051), 5, 10-methodology-THF (C00143), etc. These substances were not found in the culture medium. On the contrary, pantothenic acid, anthranilic acid and other nitrogen-containing substances had obvious up-regulation, but whether these up-regulation nitrogen-containing substances were related to glycine metabolism pathways of strains needs further follow-up.

### Nitrogen balance by heterotrophic nitrification and aerobic denitrification

The nitrogen balance calculation (Table 3) revealed that both the fungus XTY1 and the bacterium GS2 were able to transform glycine (83.6 and 82.8%), ammonium (41.3 and 82.0%) and nitrate (46.5 and 58.7%). As shown in Table 3, both strains produced and released N_2_O and N_2_ during nitrification and denitrification. However, the amount of N_2_O produced during heterotrophic nitrification of N_org_ was much lower than that produced by aerobic denitrification (36.2 and 30.6%), indicating that N_2_O was produced by NO_3_^−^ *via* denitrification.

**Table 3:** Nitrogen balance after 24 h of heterotrophic nitrification and denitrification processes by XTY1 and GS2.

Nitrogen source	XTY1					GS2				
	Utilized TN (%)				Unutilized TN (%)	Utilized TN (%)				Unutilized TN (%)
	CN	N_2_O	N_2_	X		CN	N_2_O	N_2_	X	
C_2_H_5_NO_2_	78.80	0.18	3.11	1.47	16.44	63.72	0.28	8.73	10.10	17.17
(NH_4_)_2_SO_4_	36.02	1.57	3.11	0.56	58.74	67.87	1.17	8.34	4.65	17.97
NH_2_OḤHCl	–	–	–	–	100.00	–	–	–	–	100.00
NaNO_2_	–	–	–	–	100.00	–	–	–	–	100.00
KNO_3_	6.00	36.19	3.12	1.18	53.51	11.52	30.63	8.49	8.10	41.26

X was unknown nitrogen; TN, total nitrogen; CN, intracellular nitrogen. The above values were obtained from the percentage of the component in the total nitrogen content after 24 h.

During heterotrophic nitrification of N_org_, the fungus XTY1 and the bacterium GS2 produced 94.3 and 63.7% of the total nitrogen concentration of intracellular nitrogen (CN), respectively. This result indicated that both strains transformed most of N_org_ into CN by assimilation during heterotrophic nitrification, which is consistent with *Acinetobacter* sp. JR1 transforming part of the nitrogen source into CN (Yang et al., 2019). This pathway was also demonstrated in a study by Joo et al. (2005) on the denitrification efficiency of *Alcaligenes faecalis* No. 4 under high ammonium concentrations. In that study, almost all ammonium was removed after 93 h of inoculating the strain when the initial ammonium mass concentration was up to 1,200 mg·L^−1^. Fifty percent of this was transformed to intracellular nitrogenous material, 3% was transformed to nitrification products, and 40 to 50% underwent denitrification. In a study by Chen and Ni (2012) on the nitrogen balance of *Agrobacterium* LAD9 during heterotrophic nitrification and aerobic denitrification, the initial mass concentration of total nitrogen (TN) was 7.2 mg·L^−1^. After 24 h of inoculation with this bacterium, 50.1% of TN was transformed to gaseous nitrogen compounds and 40.8% was also transformed to CN.

The remaining small fraction of N_org_ was eventually transformed to N_2_ *via* aerobic denitrification, but the specific intermediates were still unclear because they did not pass through NH_2_OH and NO_2_^−^. Further experiments using methods such as ^15^N labeling and metabolomics are needed to determine the specific metabolic pathway used by the strains.

## Conclusion

Our results with fungus XTY1 and heterotrophic nitrifying bacterium GS2 revealed that:

1. XTY1 and GS2 had different transformation capacities to different nitrogen sources, and the heterotrophic nitrification capacities of organic nitrogen were stronger (the transformation rates were more than 80%), but the nitrification capacity of XTY1 to ammonium nitrogen (41.3%) was lower.
2. The organic nitrogen transformation intermediates of the two strains were free of hydroxylamine and nitrite. More than 60% of the total nitrogen were transformed to intracellular nitrogen, and less than 0.3% to N_2_O.
3. Compared with the founded heterotrophic nitrifying strains, the two strains screened in this study may have novel metabolic pathways.
4. In future studies, methods such as ^15^N labeling and metabolomics could be used to track the metabolite characteristics of the two strains and explore their N_org_ metabolism pathways.

## Data availability statement

The datasets presented in this study can be found in online repositories. The names of the repository/repositories and accession number(s) can be found at: https://www.ncbi.nlm.nih.gov/genbank/, MW132885 https://www.ncbi.nlm.nih.gov/genbank/, MW126534.

## Author contributions

TX, LB, and ZP contributed to conceptualization and design of the study. TX and SS performed the lab work and analyzed the data. TX wrote the first draft of the manuscript. LB, BR, JY, and JL contributed to writing, review and editing. LB contributed to funding acquisition. All authors have read and approved to the published version of the manuscript.

## Funding

This research was financially supported by Liaoning Provincial Science and Technology Plan Projects (2020JH1/10300006) in China.

## Conflict of interest

The authors declare that the research was conducted in the absence of any commercial or financial relationships that could be construed as a potential conflict of interest.

## Publisher’s note

All claims expressed in this article are solely those of the authors and do not necessarily represent those of their affiliated organizations, or those of the publisher, the editors and the reviewers. Any product that may be evaluated in this article, or claim that may be made by its manufacturer, is not guaranteed or endorsed by the publisher.

## Footnotes

^1^ https://www.genome.jp/kegg-bin/show_path?Map00260

## References

[ref1] Bai S. N. Xu T. Y. Liu Y. Wang X. H. Bai L. (2018). Isolation and screening of heterotrophic nitrifying bacteria from lawn soil and characterization of nitrification products. J. Hortic. 45, 1338–1346. doi: 10.16420/j.issn.0513-353x.2017-0773

[ref2] Boer W. D. Kowalchuk G. A. (2001). Nitrification in acid soils: micro-organisms and mechanisms. Soil Biol. Biochem. 33, 853–866. doi: 10.1016/S0038-0717(00)00247-9

[ref3] Brierley E. D. R. Wood M. (2001). Heterotrophic nitrification in an acid forest soil: isolation and characterisation of a nitrifying bacterium. Soil Biol. Biochem. 33, 1403–1409. doi: 10.1016/S0038-0717(01)00045-1

[ref4] Chen Q. Ni J. R. (2012). Ammonium removal by *agrobacterium* sp. LAD9 capable of heterotrophic nitrification-aerobic denitrification. J. Biosci. Bioeng. 113, 619–623. doi: 10.1016/j.jbiosc.2011.12.012, PMID: 22296870

[ref5] Chen M. X. Wang W. C. Feng Y. Zhu X. H. Zhou H. Z. Tan Z. L.. (2014). Impact resistance of different factors on ammonia removal by heterotrophic nitrification-aerobic denitrification bacterium *Aeromonas* sp. HN-02. Bioresour. Technol. 167, 456–461. doi: 10.1016/j.biortech.2014.06.001, PMID: 25006021

[ref6] Clescerl L. S. (1998). Standard Methods for the Examination of Water and Wastewater 20th. Washington: APHA.

[ref7] Frear D. S. Burrell R. C. (1955). Spectrophotometric method for determining hydroxylamine reductase activity in higher plants. Anal. Chem. 27, 1664–1665. doi: 10.1021/ac60106a054

[ref8] Huang X. J. Xu Y. He T. X. Jia H. J. Feng M. Xiang S. D.. (2019). Ammonium transformed into nitrous oxide via nitric oxide by *pseudomonas putida* Y-9 under aerobic conditions without hydroxylamine as intermediate. Bioresour. Technol. 277, 87–93. doi: 10.1016/j.biortech.2019.01.040, PMID: 30660065

[ref9] Joo H. S. Hirai M. Shoda M. (2005). Characteristics of ammonium removal by heterotrophic nitrification-aerobic denitrification by *Alcaligenes faecalis* no. 4. J. Biosci. Bioeng. 100, 184–191. doi: 10.1263/jbb.100.184, PMID: 16198262

[ref10] Li C. E. Yang J. S. Wang X. Wang E. T. Li B. Z. He R. X.. (2015). Removal of nitrogen by heterotrophic nitrification–aerobic denitrification of a phosphate accumulating bacterium *pseudomonas stutzeri* YG-24. Bioresour. Technol. 182, 18–25. doi: 10.1016/j.biortech.2015.01.100, PMID: 25668754

[ref11] Liu Y. X. Hu T. T. Jing Z. Lv Y. K. Ren R. P. (2016). Simultaneous removal of carbon and nitrogen by mycelial pellets of a heterotrophic nitrifying fungus-*Penicillium* sp. L1. J. Biosci. Bioeng. 123, 223–229. doi: 10.1016/j.jbiosc.2016.08.009 27686594

[ref12] Paul J. P. Williams B. L. (2005). Contribution of α-amino N to extractable organic nitrogen (DON) in three soil types from the Scottish uplands. Soil Biol. Biochem. 37, 801–803. doi: 10.1016/j.soilbio.2004.09.011

[ref13] Pedersen H. Dunkin K. A. Firestone M. K. (1999). The relative importance of autotrophic and heterotrophic nitrification in a conifer forest soil as measured by ^15^N tracer and pool dilution techniques. Biogeochemistry 44, 135–150. doi: 10.1007/BF00992975

[ref14] Ren J. L. Bai X. Y. Liu Y. C. Huang X. (2021). Simultaneous nitrification and aerobic denitrification by a novel isolated *Ochrobactrum anthropi* HND19. Bioresour. Technol. 340:125582. doi: 10.1016/j.biortech.2021.125582 34332445

[ref15] Robertson L. A. Kuenen J. G. (1983). *Thiosphaera pantotropha* gen. Nov. sp. nov., a Facultatively anaerobic, Facultatively autotrophic Sulphur bacterium. J. Gen. Microbiol. 129, 2847–2855. doi: 10.1099/00221287-129-9-2847

[ref16] Rout P. R. Bhunia P. Dash R. R. (2017). Simultaneous removal of nitrogen and phosphorous from domestic wastewater using *Bacillus cereus* GS-5 strain exhibiting heterotrophic nitrification, aerobic denitrification and denitrifying phosphorous removal. Bioresour. Technol. 244, 484–495. doi: 10.1016/j.biortech.2017.07.186, PMID: 28803098

[ref17] Schmidt C. S. Richardson D. J. Baggs E. M. (2011). Constraining the conditions conducive to dissimilatory nitrate reduction to ammonium in temperate arable soils. Soil Biol. Biochem. 43, 1607–1611. doi: 10.1016/j.soilbio.2011.02.015

[ref18] Serna M. D. Pomares F. (1992). Evaluation of chemical indices of soil organic nitrogen availcapacity in calcareous soils. Soil Sci. Soc. Am. J. 56, 1486–1491. doi: 10.2136/sssaj1992.03615995005600050025x

[ref19] Streeter T. C. Bol R. Bardgett R. D. (2015). Amino acids as a nitrogen source in temperate upland grasslands: the use of dual labelled (^13^C, ^15^N) glycine to test for direct uptake by dominant grasses. Rapid Commun. Mass Spectrom. 14, 1351–1355. doi: 10.1002/1097-0231(20000815)14:15<1351::AID-RCM23>3.0.CO;2-9 10920354

[ref20] Wu M. R. Hou T. T. Liu Y. Miao L. L. Ai G. M. Ma L.. (2021). Novel *alcaligenes ammonioxydans* sp. nov. from wastewater treatment sludge oxidizes ammonia to N_2_ with a previously unknown pathway. Environ. Microbiol. 23, 6965–6980. doi: 10.1111/1462-2920.15751, PMID: 34581470

[ref21] Yang J. R. Wang Y. Chen H. Lyu Y. K. (2019). Ammonium removal characteristics of an acid-resistant bacterium *Acinetobacter* sp. JR1 from pharmaceutical wastewater capable of heterotrophic nitrification-aerobic denitrification. Bioresour. Technol. 274, 56–64. doi: 10.1016/j.biortech.2018.10.052 30500764

[ref22] Yu X. Li D. J. Li D. Zhang G. H. Zhou H. S. Li S.. (2020). Enhanced wet deposition of water-soluble organic nitrogen during the harvest season: influence of biomass burning and in-cloud scavenging. J. Geophys. Res. Atmos. 125:e2020JD032699. doi: 10.1029/2020jd032699

[ref23] Zhang M. X. Li A. Z. Yao Q. Wu Q. P. Zhu H. H. (2020). Nitrogen removal characteristics of a versatile heterotrophic nitrifying-aerobic denitrifying bacterium, *pseudomonas bauzanensis* DN13-1, isolated from deep-sea sediment. Bioresour. Technol. 305:122626. doi: 10.1016/j.biortech.2019.122626, PMID: 32143020

[ref24] Zhang J. B. Müller C. Zhu T. B. Cheng Y. Cai Z. C. (2011). Heterotrophic nitrification is the predominant NO_3_^−^ production mechanism in coniferous but not broad-leaf acid forest soil in subtropical China. Biol. Fertil. Soils 47, 533–542. doi: 10.1007/s00374-011-0567-z

[ref25] Zhang J. B. Sun W. J. Zhong W. H. Cai Z. C. (2014). The substrate is an important factor in controlling the significance of heterotrophic nitrification in acidic forest soils. Soil Biol. Biochem. 76, 143–148. doi: 10.1016/j.soilbio.2014.05.001

[ref26] Zhao B. An Q. He Y. L. Guo J. S. (2012). N_2_O and N_2_ production during heterotrophic nitrification by *Alcaligenes faecalis* strain NR. Bioresour. Technol. 116, 379–385. doi: 10.1016/j.biortech.2012.03.113, PMID: 22534373

